# Design and Implementation of Personalized Push Service Based on Feature Extraction and Pattern Recognition

**DOI:** 10.1155/2022/2688602

**Published:** 2022-07-22

**Authors:** Moxuan Zhang

**Affiliations:** Department of Mechanical Engineering, Tianjin Renai College, Tianjin, China

## Abstract

Personalized push service is one of the more popular research and application fields, which has received more and more attention. Its application prospects are also more and more extensive. This research mainly designs and implements personalized push services through feature extraction and pattern recognition. In this study, the Chinese texts of user-visited pages are classified according to keywords, so as to obtain the user's interest characteristic data. Then, according to the frequency of each feature category, the weight of the user's interest feature is calculated, and the user's interest field is predicted and identified. After that, resources that match the user's interest field are pushed to it. In order to verify the effectiveness of the improved model, this study carried out experiments and comparisons on the precision rate, recall rate, and comprehensive classification rate of the original model and the improved model on the implemented personalized push service system. In the research, the error between the interest results under each interest topic in the test set and the results obtained by the statistical analysis of the training set is within a reasonable range, the maximum of which is about 5%. The accuracy of interest degree prediction in different scenarios can reach more than 90%, which directly confirms the good applicability and effectiveness of the analysis and calculation method and the constructed model for user interest in this study. The personalized push service framework proposed in this study has good application value in the field of time-sensitive information services.

## 1. Introduction

The personalized push service can analyze and extract the user's interest from the collected user-related information in order to further understand the user. In addition, user behavior analysis can explore the behavior characteristics and information needs of users. In this way, resource information that matches user interests can be discovered, and it can be actively pushed to users to meet their differentiated needs of users. Personalized push service is the concrete embodiment of information personalized service. With the rapid development of computer technology, computers with large memory and fast and accurate computing have appeared in our real life, which makes people's ways of obtaining information resources develop towards diversification. The user's basic information, interests, usage behavior, and other information are continuously collected by the computer, and different databases are established for different users, thereby truly providing different personalized push services for different users. With the development of network technology, users can also have different personalized page customization when using the personalized push system. Users can also choose different types of services such as resources they need according to their actual situation.

According to the different needs of different users, it is very necessary to provide personalized push services, change passive services into active services, and assist users to filter out accurate and useful information from a large amount of data. Wohllebe et al. believed that push notifications are the core function of mobile applications [1]. Lyu et al. proposed a new model called deep matching to ranking (DMR) [2]. Xiao-Long et al. proposed a task-oriented multicollaborative filtering algorithm [3]. Wang et al. proposed a collaborative filtering algorithm recommendation system based on device-edge-cloud federated learning [4]. Ting proposed two data dissemination models: a data pull model in which mobile users acquire data from data providers [5]. Their research precision on personalized push services is not very high. Therefore, this paper reviews relevant materials and decides to use feature extraction pattern recognition to optimize personalized push services.

Most of the current systems obtain their personal information through the way of user submission and lack the ability to track user behavior and actively analyze their behavior and extract user features to extract pattern recognition, so the personalized information inside the system is static. Jenke et al. believed that emotion recognition can assess the user's state [6]. Borges et al. proposed a method aimed at extracting features to obtain disturbance information related to power quality [7]. Matthew et al. used micro-Doppler signals collected by multistatic radar to detect and distinguish hovering and flying micro-UAVs carrying different payloads [8]. Pei et al. proposed a SAR ATR feature extraction method based on two-dimensional principal component analysis [9]. In order to reduce the cost of labeling samples, Liu et al. proposed a new semisupervised algorithm (SNC) with neighborhood constraints [10]. Using feature extraction pattern recognition, it is necessary to establish feature constraints based on user attributes and user interest-based feature constraints. The feature extraction pattern recognition will be further explored in the following sections.

The identification of information features mainly requires users to actively submit real data that can reflect their preferences to the system. The data provided by the user mainly includes the user's personal attribute information, as well as the subjective display score and text evaluation made by the user. This paper analyzes and constructs the push service architecture. It also designs each module of the architecture separately, including five modules: log file preprocessing, user behavior feature analysis, literature resource feature analysis, information push, and real-time data update. Instances are verified for the personalized push service architecture. That is, taking a user as an example and analyzing the user's behavior log, interest features are extracted. Then, information resources consistent with their interest features are pushed. This proves the rationality and feasibility of the architecture. Both push service and web page advertisement push need to analyze user behavior and extract users' interest characteristics, so as to predict their areas of interest and push relevant information resources to them. When the number of neighbors is greater than 28, the recommendation accuracy of the feature extraction pattern recognition model in this study is the highest. When the number of neighbors is less than 28, in terms of recommendation accuracy, the recommendation accuracy of the feature extraction pattern recognition model proposed in this study is better than the recommendation accuracy of the *K*-means algorithm and Pearson correlation.

## 2. Design and Implementation of Personalized Push Service

### 2.1. Personalized Push Service

Personalized push service actively sends information resources matching their interest characteristic data to users in a purposeful and timely manner according to user needs. A personalized push service is suitable for all kinds of users, and it can push continuously updated information resources to users. The personalized push service has the following characteristics:

1. Differences According to Users. The personalized push service is user-centric. On the basis of fully mining and researching the user's browsing habits, search, borrowing records, and other information, according to the user's personal needs, it will discover the resource information that matches the user's information needs and actively push the information to users in order to meet the needs of different users [11].
2. Initiative. Initiative is the most fundamental characteristic of personalized push service. Personalized push service has changed the previous mode of passively providing information to users. Instead, the digital library actively pushes information resources to users. When new users or new resources appear, or when user behavior logs are updated, the library will analyze the characteristic attributes of relevant information resources for users to consult anytime, anywhere.
3. Efficiency. Through personalized push service, users can obtain the information resources they need from massive information resources, which helps to improve the efficiency of user resource acquisition. In addition, when the information resources are updated, the resources will be analyzed and actively pushed to the users in need, thus avoiding the waste of user time.

Digital resource integration is an important aspect of personalized push services. Resource integration can integrate not only various types of resources, cluster, or classify resources but also related data resources together, so as to provide users with the appropriate information, shortening the time for users to search for information resources, which provides users with efficient and personalized services. Through the matching of users and resources, the most interesting information can be recommended to user groups with similar interests [12].

### 2.2. Feature Extraction Pattern Recognition

The foundation of the user's interest model is the extraction of data features, and the recommendation system uses these data features as the basis for extracting user interests. The key to building a model is the representation method and structural feature information of the model, which directly relate to the availability and computability of the model. This research adopts the method of feature extraction pattern recognition to develop personalized push service and also constructs the key points of user portrait and personalized extraction.

The simplest Boolean expression is equality, which tests whether one value is the same as another. The Boolean weight (binary) is based on whether the feature word appears in the text as the basis for weight assignment, and its formula is expressed as

(1) $$\begin{array}{c} W_{ij}=\left\{\begin{array}{cc} 1, & {\text{TF}}_{ij}>0\\ 0, & {\text{TF}}_{ij}\leq 0 \end{array}\right., \end{array}$$

where *W*_*ij*_ represents the weight of feature word *i* in text *j*, and TF_*ij*_ represents the number of times feature word *i* appears in text *j* [13].

TF/IDF is used to evaluate the importance of a word to a text in the training set. Among them, TF represents the frequency of a certain word in the text, that is, the word frequency. The idea of the TF weight calculation method is that the frequency of feature words appearing in the text is proportional to its importance. That is, the higher the frequency of occurrence is, the more important the feature word will be in the text, and the greater its weight will be. Its calculation formula is as follows:

(2) $$\begin{array}{c} W_{ij}={\text{TF}}_{ij}. \end{array}$$

The calculation method of the membership degree of the text to be classified in each category is as follows:

(3) $$\begin{array}{c} \text{score}\left(d_{i},C_{j}\right)=\sum_{i,j=1}^{k}\text{sim}\left(d_{i},d_{j}\right)y\left(d_{i},C_{j}\right), \end{array}$$

where *y*(*d*_*i*_, *C*_*j*_) is the classification value of the text pair category in the training set, and its value range is {0, 1} [14].

Given the mother wavelet function *ψ*(*t*), on the continuous (*a*, *b*), the wavelet transform basis function can be defined in the time-frequency space as

(4) $$\begin{array}{c} \Psi_{ab}\left(t\right)=\frac{1}{\sqrt{a}}\Psi \left(\frac{t-b}{a}\right)↔\phi_{ab}\left(\Omega \right)=\sqrt{a}\phi \left(a\Omega \right){\text{e}}^{-jb\Omega }. \end{array}$$

The inverse transform of the wavelet transform of the signal can be defined as

(5) $$\begin{array}{c} f\left(t\right)=\frac{1}{C_{\Psi }}\overset{+\infty }{\underset{-\infty }{\int }}\overset{+\infty }{\underset{0}{\int }}\frac{\text{d}a\text{d}b}{a^{2}}W^{f}a_{0}\left(a,b\right)\Psi_{ab}\left(t\right). \end{array}$$

The scale function also satisfies the orthogonal condition when the scale parameters are equal [15].

(6) $$\begin{array}{c} \int^{}\phi_{mn}\left(t\right)\phi_{ms}\left(t\right)\text{d}t=\delta_{n-s}. \end{array}$$

The set of scaling functions can be defined as [16]

(7) $$\begin{array}{c} \left\{\phi_{mn}\left(t\right)\right\}=2^{-\left(m/2\right)}\phi \left(2^{-m}t-n\right). \end{array}$$

In CRP, the local compactness of a sample is characterized by the following minimization problem [17]:

(8) $$\begin{array}{c} \underset{P}{\min}\sum_{i=1}^{n}P^{T}x_{i}-\sum_{j=1}^{n}s_{j,i}P^{T}x_{j}=tr\left(P^{T}S_{L}P\right). \end{array}$$

On the other hand, the maximization of the sample population divergence can be expressed as [18]

(9) $$\begin{array}{c} \underset{P}{\text{max}}\sum_{i=1}^{n}P^{T}x_{i}-P^{T}m_{2}^{2}=tr\left(P^{T}S_{t}P\right), \end{array}$$

where *m* is the population mean of all samples [19].

The formula for the predicted score is as follows [20]:

(10) $$\begin{array}{c} P_{u,k}=\bar{R_{k}}+\frac{\sum_{i\in I}\text{sim}\left(k,i\right)\left(R_{u,i}-\bar{R_{i}}\right)}{\sum_{i\in I}\left(\left|\text{sim}\left(k,i\right)\right|\right)}, \end{array}$$

where *P*_*u*,*k*_ represents the predicted score of user *U* for item *k*.

The calculation formula of the weighted slope one algorithm is as follows:

(11) $$\begin{array}{c} P_{w}{\left(u\right)}_{j}=\frac{\sum_{i\in R_{j}}\left({\text{dev}}_{j,i}+u_{j}\right)\text{card}\left(S_{j,i}\right)}{\sum_{i\in R_{j}}\text{card}\left(S_{j,i}\right)}, \end{array}$$

where *P*_*w*_(*u*)_*j*_ represents the predicted rating of item *j* by target user *u*.

According to the Euclidean distance, the attribute similarity of *i* and *j* can be calculated, and the calculation formula is as follows:

(12) $$\begin{array}{c} {\text{sim}}_{i}\left(i,j\right)=\frac{1}{1+d\left(i,j\right)}. \end{array}$$

Through the attribute similarity between items, the weighted sum method is used to calculate the user's predicted score for the item. The weighted average is to multiply each value by the corresponding weight, then add and sum to get the overall value, and finally divide by the total number of units. The specific calculation formula is as follows:

(13) $$\begin{array}{c} u_{i}=\frac{\sum_{j=1}^{n}u_{j}*{\text{sim}}_{s}\left(i,j\right)}{\sum_{j=1}^{n}{\text{sim}}_{s}\left(i,j\right)}. \end{array}$$

Manhattan distance represents the nonlinear distance between two data objects. The formula is as follows:

(14) $$\begin{array}{c} d\left(x_{i},x_{j}\right)=\overset{m}{\underset{k=1}{\sum }}\left|x_{ik}-x_{jk}\right|. \end{array}$$

The variance weighted distance is as follows:

(15) $$\begin{array}{c} d\left(x_{i},x_{j}\right)={\left[\overset{m}{\underset{k=1}{\sum }}\frac{{\left(x_{ik}-x_{jk}\right)}^{2}}{s_{k}^{2}}\right]}^{1/2}. \end{array}$$

Euclidean distance formula is as follows:

(16) $$\begin{array}{c} d\left(x_{i},x_{j}\right)=\overset{m}{\underset{k=1}{\sum }}\frac{{\left(x_{ik}-x_{jk}\right)}^{2}}{s_{k}^{2}}. \end{array}$$

The information entropy formula of information source *X* is as follows:

(17) $$\begin{array}{c} H\left(X\right)=\sum_{i=1}^{n}p_{i}\log_{2}\frac{1}{p_{i}}, \end{array}$$

where *n* indicates that there are *n* categories in the sample set *X*, and *p*(*ai*) indicates the frequency of the *i*-th category in the sample set *X*. The formula of the weighted information entropy of user *U*_*m*_ and user *U*_*n*_ is as follows:

(18) $$\begin{array}{c} \text{JWDE}\left(U_{m},U_{n}\right)=-\frac{2n}{N1+N2}\sum_{i=1}^{N}p\left(d_{i}\right)\log_{2}\left(p\left(d_{i}\right)\right)\times \left|d_{i}\right|. \end{array}$$

According to the similarity between user *m* and its nearest neighbors, the classical nearest neighbor prediction method is used to predict the score of *m* on unknown items as follows:

(19) $$\begin{array}{c} P_{m,J}=\bar{R_{m}}+\frac{\sum_{i\in \text{NBS}}\text{Sim}\left(m,i\right)\left(R_{I,J}-\bar{R_{I}}\right)}{\sum_{i\in \text{NBS}}\left|\text{Sim}\left(m,i\right)\right|}. \end{array}$$

### 2.3. Personalized Push Service Architecture

**Figure 1:**
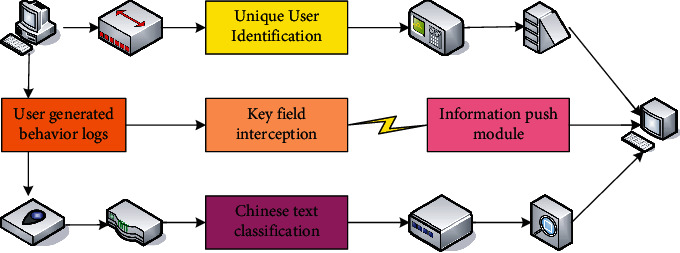
Architecture design of personalized push service.

The architectural design of the personalized push service is shown in Figure 1. First, the behavior data (log files) generated is preprocessed during the user's use. Then, the content of the resource page accessed by the user is captured by the web crawling technology, and the Chinese text word segmentation is performed on the page. Then, the weight of each feature word is calculated, so as to extract the weighted keywords. According to the keywords, the Chinese texts of the pages visited by the users are classified in order to obtain the user's interest characteristic data. Next, according to the frequency of occurrence of each feature category, the weight of the user's interest feature is calculated. The user's interest field is predicted, and then, resources that match the user's interest field can be pushed to it.

#### 2.3.1. Design of Web Service Architecture

Web service is a distributed computing technology, also known as a platform-independent technology, which is used to integrate and connect applications on various platforms. When the mobile terminal needs to call the service, it needs to find out which server can provide such service on UDDI. UDDI is a technology for describing, discovering, and integrating web service, which is an important part of the web service protocol stack. After finding the server, the mobile terminal asks for the specific calling method, and the server needs to provide the specific method interface of the service according to the method. That is, it returns an XML document described in WSDL format, which describes the interface and its parameters and return type. The mobile terminal uses SOAP to encapsulate the content according to the interface description and sends an HTTP request. Then, the server returns a SOAP packet. In this way, requests and responses at both the server and the mobile end can be unblocked. When the ESB (the full name of ESB is enterprise service bus, which generally refers to enterprise service bus) receives the request message, it first looks up the requested service in the internal service registry and converts the transmission protocol and message format in the request message according to the service needs. Then, it delivers the message to the service provider according to the service path. After obtaining the returned result, the ESB will again convert the message format or transmission protocol to the type supported by the service requester, finally sending the result to the service requester.

#### 2.3.2. User Login Interface Settings

The user needs to enter the personalized push system through the user interface of the login system, which is the foreground program for the user to perform various operations. Through the research and analysis of the personalized push service objects, the user authentication module of the system is designed according to the level of service required by users. Different levels of users have different needs for services. High-level users may need some highly targeted services, while low-level users may only need some simple and rudimentary services. When designing the landing module, different landing page settings should be made according to the different needs of users. In the system, when the user logs in, a window for whether to customize the service will appear, and the user can choose after authentication. If customized information is required, the system will automatically extract the user's registration information from the user's information database and then match it according to the user's preferences. Then, the user is taken to a page with customized information. If the user does not need to customize the information, the system will automatically enter a relatively simple personalized service interface.

#### 2.3.3. Information Push Interface Design

**Figure 2:**
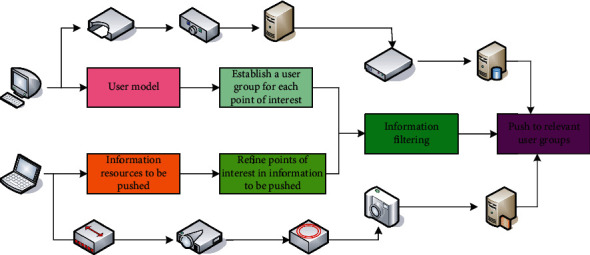
Specific information push.

Personalized push service is a service method with strong initiative and individuality. The information push interface is an important part of the system. This personalized push method is realized by using the relevant theories and technologies of complex networks. The establishment of the user model is dynamic, and the user's information base can be updated in real time with the change in readers' interests. Therefore, the accuracy of the information push is greatly improved. The service quality of the information push is also improved. The specific information push is shown in Figure 2. The information resources to be pushed are clustered according to different subject words. The resources with the same point of interest are divided into one group, and the information resources of different points of interest are divided into different groups. After clustering, several information resource groups with common points of interest within groups and different points of interest among groups are formed.

#### 2.3.4. Data Storage and Interface Design

The services layer is added to the infrastructure to interact directly with the database while reducing the frequency of the controller accessing the model directly, which can reduce coupling and facilitate expansion. In addition, how to choose a good data storage method is very important for an application that needs to display data frequently. Meanwhile, for the interface that frequently displays data, memory management is very important. In addition to using the ARC technology recommended by iOS, the timing of data acquisition and interface refresh, the reuse of cells, and the addition and deletion of subviews must be carefully grasped. Cell array integrates related data of different types into a single variable, making it easy and convenient to reference and process a large amount of related data.

#### 2.3.5. Design and Implementation of Application Push Module

The data source of the push module mainly consists of three parts: user personal information, application basic information, and user usage of the application. Collection and processing of user personal information refer that user personal information is obtained through telecom's CRM management system, mainly including the user's name, age, and gender. Large-scale customer relationship management system (CRM) is a computer management system for business processing, operation management, and customer service by telecom key customer management departments. The push module will store this information in a database for later use. The basic information of the application is obtained in the application management module, including the name, category, trigger action, and application icon of the application. In this system, three attributes of application category, delivery time, and trigger action are selected as the characteristics of the project. According to these three attributes, the item attribute matrix is established to calculate the similarity. In terms of measuring user usage of the application, this paper focuses on how users interact with the application through the application's user interface. According to the RFD model, the user's score for the application is obtained as shown in the following formula:

(20) $$\begin{array}{c} {\text{score}}_{u,i}=\alpha S_{R}+\beta S_{F}+\gamma S_{D}, \end{array}$$

where score_*u*,*i*_ represents the user's score for an application (i.e., the user's preference for the application). *S*_*R*_ represents the time difference between the user's last use of the app and the current time. *S*_*F*_ is the number of times the user clicked the app. *S*_D_ represents the total time the user interacts with the application.

#### 2.3.6. Design and Implementation of Client Software

The application package receiver is mainly responsible for monitoring whether the user has installed or uninstalled an application. User behavior monitors are used to record the number and duration of user interactions with the application. The user behavior information uploader will upload the user behavior records to the server at regular intervals. Requests for downloading applications and uploading data are placed in the request queue, and the task executor processes these tasks by issuing HTTP requests to the server. Then, the application converts into the state running in the foreground. If the user starts another application, the current application loses focus and converts into the background running state. Once the user presses the navigation button or the screen unlock key, the application returns to the foreground running state. When it is terminated by the user or terminated by the android operating system (such as resource recycling), the application will return to the stopped state. If some service center applications may always run in the background, their status will directly change from the stopped state to the background running state.

The personalized push service architecture designed in this paper includes data storage module, information push module, and user login module. The data source of the push module mainly includes three parts: user personal information, application basic information, and user usage of the application. This article focuses on how users interact with an application through the application's user interface.

## 3. Personalized Push Service Design Results

Data operations such as RSS information storage, user interest model storage, user interest model update storage, and recommendation information storage must be completed by the database. According to the functional requirements of the system, the main database relationships designed using SQL Server 2005 database tools are shown in Table 1.

After receiving the request, the public open platform sends the information to the distribution module. Then, the distribution module will store the information in Table 4-luser__device_token database table. It mainly contains 8 fields, of which the primary key is the unique identifier of the user's device. The user ID is the user's ID information. The application ID is the application ID number applied by the merchant in the management console. The device token is the unique device identifier of the app user's mobile phone. The device type is the operating system type of the user's mobile phone. User extension information is the information customized by the merchant, such as avatar and nickname which is in json format. Creation time is the creation time of this record, and the update time is the update time of this field. The basic information of users is shown in Table 2.

According to user access characteristics, all users can be divided into four types: (1) users with stable interests and narrow interests; (2) users with stable interests and wide interests; (3) users with unstable interests and narrow interests; (4) users whose interests are unstable and wide-ranging. Representative users A, B, C, and D are selected from the four different user types for testing, and the number of users A, B, C, and D using the recommended function is 10. The total amount of information obtained is the same. The test results of the four types of users using the original model are shown in Table 3.

**Figure 3:**
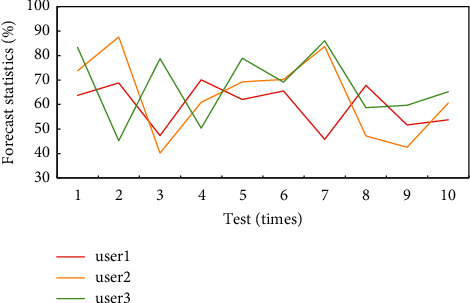
Interest degree prediction statistics.

The statistics of interest degree prediction are shown in Figure 3. During the weekdays, the first user was most interested in clothing and digital goods, while on weekends his interest turned to shoes and food. The second user stopped paying too much attention to digital at the weekend, and his interest in home appliances increased greatly. The third user became more interested in food at the weekend and began to pay attention to digital goods at the same time. Therefore, the user's interest level is often closely related to the situation. By analyzing the situation, the user's interest can be more accurately grasped, which is also more conducive to the precise marketing of service providers. The error between the interest results under each interest topic in the test set and the results obtained from the previous statistical analysis of the training set is within a reasonable range, and the maximum is about 5%. The accuracy of interest degree prediction in different scenarios can reach more than 90%, which directly confirms the good applicability and effectiveness of the analysis and calculation method and the constructed model of user interest in this paper.

**Figure 4:**
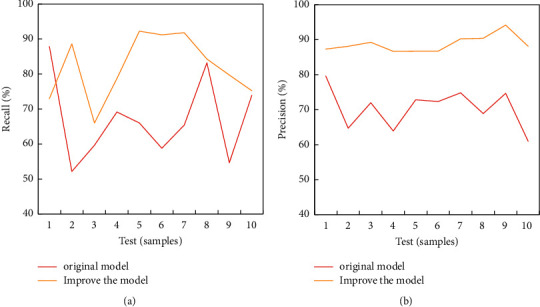
Comparison of using the original model and the improved model. (a) Recall ratio. (b) Precision ratio.

For users with unstable interests, the recall rate is not high; for users with wide interests, the precision rate is not high (the recall rate is shown in Figure 4(a)). The improved model can improve the recall rate and precision rate of the four types of users, and the comprehensive classification rate of the four types of users is also improved, especially for users with unstable interests and wide interests. This shows that the improved method of the user interest model proposed in the study is feasible (the precision rate is shown in Figure 4(b)).

The common push agent stores the crawled news web pages in the local directory corresponding to the source website. Then, the specific web page analysis process analyzes the structure and content of the web page and extracts the news title, text, time, category, and other information. Then, it stores them in the corresponding fields of the news table in the local database. The processing of all web pages relies on a specific analysis of a given web page. The specific judgment of the news category is carried out when the news information is stored in the database. The MetaNews relational model is shown in Table 4.

The fixed type contains the least user knowledge and has the lowest weight. The user's direct selection can most clearly reflect the user's tendency and has the highest weight. The weight values of various types of information are shown in Table 5. The reliability coefficient of each field is calculated. When the reliability coefficient of a field exceeds or falls below a certain threshold, the degree of user attention in this field will be changed. For example, when the reliability coefficient of a user's interest field is lower than 0, the field will become the user's uninteresting field.

**Figure 5:**
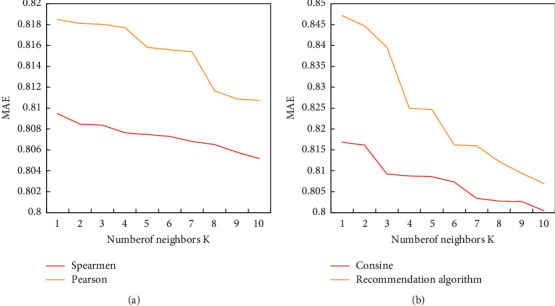
MAE values under each similarity measure. (a) Pearson correlation and Spearman correlation. (b) Cosine similarity and personalized recommendation.

The smaller the value of MAE is, the better the recommendation effect of the system will be. The user's unrated items are predicted on the test set, so that the number of nearest neighbors gradually increases with a stride of 5 (Pearson correlation and Spearman correlation are shown in Figure 5(a)).  Figure 5 shows the MAE values of the four methods of Pearson correlation, Spearman correlation (it is a nonparametric measure of the dependence of two variables, which uses a monotonic equation to evaluate the correlation of two statistical variables), cosine similarity, and personalized recommendation on different number of neighbors. Among them, the selection of the number of nearest neighbors is 13 cases such as 5, 10, 15, 20, 25, 30, 35, 40, 45, 50, 55, 60, and 65 (cosine similarity and personalized recommendation are shown in Figure 5(b)). In the improved algorithm (personalized recommendation), when the number of neighbors is 40, the value of MAE tends to be stable, and the value of MAE is always smaller than that of the other three similarity measures. The personalized recommendation method proposed in this paper can significantly reduce MAE and improve the recommendation effect of the recommendation system. In subsequent experiments, the number of nearest neighbors *k* is chosen to be 45.

**Figure 6:**
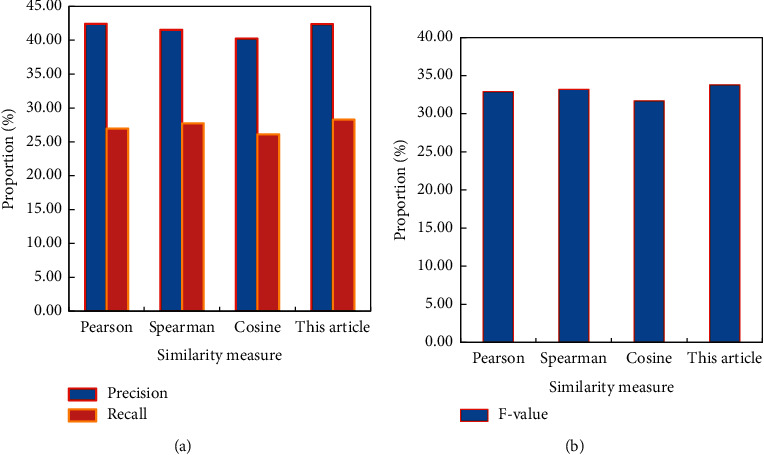
Comparison of recommendation quality of different similarity methods. (a) Precision and recall ratio. (b) *F* evaluation.

Except that the result calculated by the recommendation algorithm in the column of accuracy rate is not optimal, the other two evaluation indicators are optimal. Compared with other similarity measurement methods, it has a relatively obvious improvement (precision and recall rates are shown in Figure 6(a)). The accuracy of the recommendation algorithm is less than one-thousandth lower than the optimal value. Therefore, the recommendation algorithm has a good recommendation effect on the whole (*F* evaluation is shown in Figure 6(b)).

**Figure 7:**
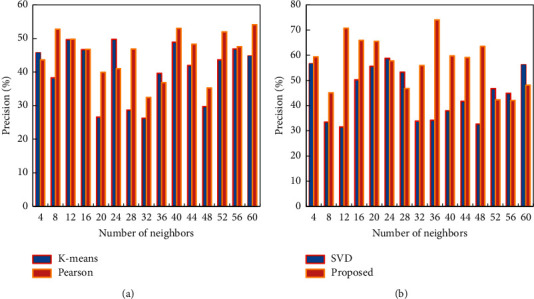
Comparison of precision values for different number of neighbors. (a) *K*-means and Pearson. (b) SVD and proposed.

When the number of neighbors is less than 20, the MAE value of the improved algorithm in this study is smaller than the MAE value obtained by the *k*-means algorithm and Pearson correlation. When it is greater than 20, the MAE value of the improved algorithm is also greater than that obtained by SVD-based collaborative filtering. In other words, when it is less than 20, the improved algorithm in this study is better than the *k*-means algorithm and the Pearson-related collaborative filtering recommendation algorithm. When it is greater than 20, the performance of the improved algorithm in this study is optimal (*K*-means and Pearson are shown in Figure 7(a)). When the number of neighbors is greater than 28, the improved algorithm in this study has the highest recommendation accuracy among the above four algorithms. When the number of neighbors is lower than 28, in terms of recommendation accuracy, the recommendation accuracy of the improved algorithm is better than that of the *K*-means algorithm and Pearson-related recommendation, which is slightly lower than the recommendation based on SVD (SVD is singular value decomposition. Its full name is singular value decomposition), but the overall recommendation effect is greater than 80%. It can be seen from the experiments that the bipartite *k*-means collaborative filtering algorithm based on SVD data dimensionality reduction proposed in this study improves the recommendation effect. Moreover, the good scalability of the algorithm is also preserved. Meanwhile, the problem of local optima generated by the algorithm is alleviated as much as possible (SVD and proposed are shown in Figure 7(b)).

**Figure 8:**
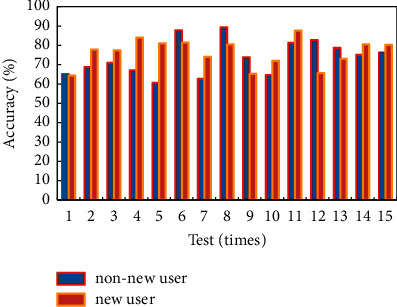
New user and nonnew user test.

The test for new users and nonnew users is shown in Figure 8. Some classmates and friends are invited to test the recommendation function of the platform offline. By simulating user registration and logging in to the platform, and entering personal information, accessing part of the website for scoring and other behaviors, the user's personal attributes and historical scoring records are formed in the system. Collection and counting user feedback on the recommended content refers to getting a list of recommended content after logging in to the platform multiple times, as well as testing whether the user is interested in the content in the list. The test content is aimed at testing the recommendation accuracy of the two recommendation strategies in the combined recommendation algorithm. The test user objects are divided into two cases: new users and nonnew users. A “new user” is a user who does not have any scoring behavior after registering and logging in. “Nonnew users” are users who have generated 20 content rating actions.

**Figure 9:**
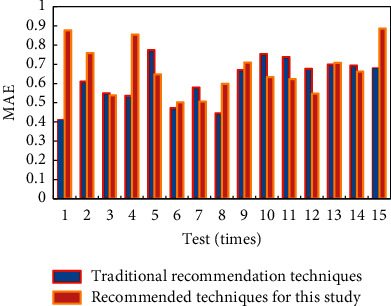
MAE value comparison.

In order to compare the performance of hybrid push technology based on interest degree and traditional collaborative filtering technology, the following experimental methods are used in this paper: 10 users are randomly selected as experimental samples, and then, the interest data of 80% of the interest topics of these 10 users are used as the known data of the samples. The interest degree of the remaining interest topics is predicted by the hybrid push technology based on interest degree and the traditional collaborative filtering technology. Finally, the predicted interest degree is compared with the real interest degree data of the other 20% of interest topics. The comparison of the mean absolute deviation MAE is used to verify the pros and cons of the two push technologies. MAE (the full name of MAE is mean absolute error, that is, the mean absolute value error, which represents the average value of the absolute error between the predicted value and the observed value) values of the sample users are averaged in each experiment. The MAE value comparison results are shown in Figure 9. The mean absolute deviation MAE directly reflects the level of recommendation accuracy. The experimental results show that the hybrid push technology based on interest degree is better than the traditional collaborative filtering technology in terms of the MAE index, which improves the overall push quality to a certain extent.

**Figure 10:**
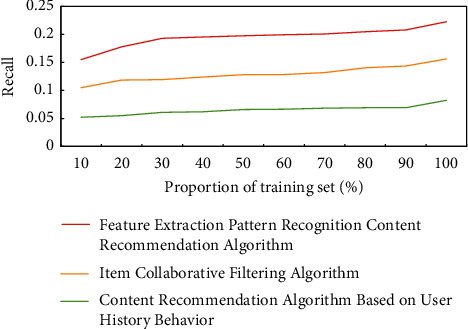
Comparison of the experimental results of the three algorithms under the hit rate index.

The comparison of the experimental results of the three algorithms under the hit rate index is shown in Figure 10. The node data are the mean of the recommendation accuracy of all test users. The hit rate in this paper refers to the ratio between the number of actual “hit” user interests in the content list recommended by the system to the user and the length of the recommended content list. In this experiment, each of the three algorithms recommends 100 pieces of content to each test user (there are more than 9,000 pieces of content in the content dataset). The percentage displayed on the abscissa refers to the proportion of the training set (e.g., 80% means that 80% of the sample data of movie reviews are used for training and 20% is used for testing). In the case of a large number of training samples, the reason for the low hit rate of the three algorithms is that the total number of valid evaluations for a single user is limited (about 100). When the number of training samples is large, the number of test samples is very small, so the number of hit test sets is small. Therefore, when the number of recommended contents remains unchanged (the convention is 100), the hit rate will increase with the reduction of training data. However, in general, the feature extraction pattern recognition content recommendation algorithm based on real-time feedback of user interests proposed in this paper is superior to the traditional mainstream item-based collaborative filtering algorithm (the collaborative filtering algorithm discovers the user's preference based on the mining of the user's historical behavior data. It can predict the products that the user may like to recommend) and user historical behavior-based content recommendation algorithm under different training sets, which is especially obvious when the training samples are few; that is, the data are sparse.

## 4. Conclusion

Generally speaking, data features are the source of analyzing interest models, which mainly refer to a series of data related to the characteristics and preferences of users. In this paper, a new user interest model is established on the basis of user interest feature information mining, including the user's personal interest set and feature extraction algorithm, so that the personal push agent has the ability to learn and track user interest. The personalized push service system designed in this paper can provide information delivery methods such as e-mail and short messages according to user needs and can be set by users according to their own conditions. The whole system has a common push agent, which is mainly responsible for the operation of various functions at the system level, and interacts, collaborates, and shares knowledge with the user's personal push agent to jointly complete the task of personalized information push. Due to the limited time, there are still some areas in the research that need to be further studied and improved. The setting of some parameters in the user interest model needs to be further studied, such as the setting of the similarity threshold and the setting of the sliding window size. These parameters have a great influence on the results and should have better adaptability. With the continuous operation of the system, the database will continue to increase. How to better organize and manage information data and optimize the user interest model is the work that needs to be further improved.

## Figures and Tables

**Table 1:** Main database relationships designed using SQL Server 2005 database tools.

Serial number	Column name	Illustrate
1	Id	ID number of the RSS feed
2	Name	The name of the RSS feed
3	Ur	The address of the RSS feed

**Table 2:** User basic information.

Frequency	Field name	Type
1	id	int
2	user_id	int
3	app_id	varchar (200)
4	device_token	varchar (200)

**Table 3:** Test results of four types of users using the original model.

Frequency	Number of information bodies of interest to users	The number of candidates for the number of information bodies recommended by the system
1	6	8
2	5	9
3	7	9
4	3	3

**Table 4:** MetaNews relational model.

Frequency	Field name	Field type	Notes
1	SiteNO	int	Site ID, also a keyword
2	SiteName	char (100)	Website name
3	IndexURL	char (200)	The address of the website is often also the URL of the news category index page
4	LocalDir	char (200)	The local storage path of the website page

**Table 5:** Various information weight values.

Source	Weights	Certainty
Fixed type	1	1
Simple documentation feedback	3	2
Result documentation feedback	2	2
Direct selection	4	1

## Data Availability

The data used to support the findings of this study are available from the corresponding author upon request.
